# Transcriptome Analysis during Human Trophectoderm Specification Suggests New Roles of Metabolic and Epigenetic Genes

**DOI:** 10.1371/journal.pone.0039306

**Published:** 2012-06-22

**Authors:** Said Assou, Imène Boumela, Delphine Haouzi, Cécile Monzo, Hervé Dechaud, Issac-Jacques Kadoch, Samir Hamamah

**Affiliations:** 1 CHU Montpellier, Institute for Research in Biotherapy, Hôpital Saint-Eloi, INSERM U1040, Montpellier, France; 2 Université MONTPELLIER1, UFR de Médecine, Montpellier, France; 3 ART-PGD department, CHU Montpellier, Hôpital Arnaud de Villeneuve, Montpellier, France; 4 Département d’Obstétrique Gynécologie, Université de Montréal, Hôpital Saint-Luc du CHUM, Montréal, Canada; University of Jaén, Spain

## Abstract

In humans, successful pregnancy depends on a cascade of dynamic events during early embryonic development. Unfortunately, molecular data on these critical events is scarce. To improve our understanding of the molecular mechanisms that govern the specification/development of the trophoblast cell lineage, the transcriptome of human trophectoderm (TE) cells from day 5 blastocysts was compared to that of single day 3 embryos from our *in vitro* fertilization program by using Human Genome U133 Plus 2.0 microarrays. Some of the microarray data were validated by quantitative RT-PCR. The TE molecular signature included 2,196 transcripts, among which were genes already known to be TE-specific (*GATA2*, *GATA3* and *GCM1)* but also genes involved in trophoblast invasion (*MUC15)*, chromatin remodeling (specifically the DNA methyltransferase *DNMT3L)* and steroid metabolism (*HSD3B1*, *HSD17B1* and *FDX1).* In day 3 human embryos 1,714 transcripts were specifically up-regulated. Besides stemness genes such as *NANOG* and *DPPA2*, this signature included genes belonging to the *NLR* family (*NALP4*, *5*, *9*, *11* and *13*), Ret finger protein-like family (*RFPL1, 2* and *3*), Melanoma Antigen family (*MAGEA1*, *2*, *3*, *5*, *6* and *12*) and previously unreported transcripts, such as *MBD3L2* and *ZSCAN4*. This study provides a comprehensive outlook of the genes that are expressed during the initial embryo-trophectoderm transition in humans. Further understanding of the biological functions of the key genes involved in steroidogenesis and epigenetic regulation of transcription that are up-regulated in TE cells may clarify their contribution to TE specification and might also provide new biomarkers for the selection of viable and competent blastocysts.

## Introduction

Pre-implantation development of mammalian embryos encompasses a series of critical dynamic events, such as the transition from a single-cell zygote to a multicellular blastocyst and the first segregation of cells within the embryo with the formation of the inner cell mass (ICM) surrounded by trophectoderm (TE) cells. ICM retains pluripotency and gives rise to the embryo proper, whereas TE cells play an important role in embryonic implantation in the uterine endometrium and placental formation. In humans, the embryonic genome activation (EGA) program is functional by day 3 after fertilization [1]. The 6–8 cell stage embryo (day 3 post-fertilization) starts the process of “compaction” that leads to the generation of the tightly organized cell mass of the morula and is followed by differentiation of the morula into a blastocyst [2]. The transition from day 3 embryos to day 5 blastocysts is likely to be controlled by many and specific changes in the expression of different genes as this process involves both cellular differentiation and transcriptional reprogramming. Although some genes that are specifically expressed in day 3 human embryos and in TE cells, such as *CCNA1* and *GATA3* respectively have been identified [3], [4], our knowledge on the changes in gene expression associated with the initial embryo-TE transition and the specification of the TE cell lineage is still limited. In addition, since TE biopsies from day 5 human blastocysts might become a reliable alternative to blastomere biopsies to assess the expression of biomarkers of embryo viability [5], a better knowledge of the genes that are specifically expressed in TE cells and the embryo proper is crucial. Recent technological advances in mRNA amplification methods and DNA microarray assays have allowed the simultaneous analysis of the transcript level of thousands of genes in one experiment, thus offering a global view of the molecular events regulating physiological functions and cellular processes [6], [7]. Indeed, these methodologies have already contributed to improving our knowledge on the genetic network controlling key stages of pre-implantation embryo development [8], [9], [10], [11]. In this study, we used high-density oligonucleotide Affymetrix HG-U133P microarray chips to analyze the gene transcription profiles of single day 3 human embryos and TE cells isolated from day 5 blastocysts. By comparing the transcriptomes of TE cells and day 3 embryos, we identified the specific molecular signature of human TE cells. These findings should provide a base for investigating the molecular mechanisms of the embryo-TE transition as well as important insights for the development of diagnostic tests to test blastocyst quality in assisted reproduction programs.

## Results

### Dynamic Changes in Overall Gene Expression in Mature MII Oocytes, Single Day 3 Embryos, TE Cells from Day 5 Blastocysts and hESCs

**Figure 1:**
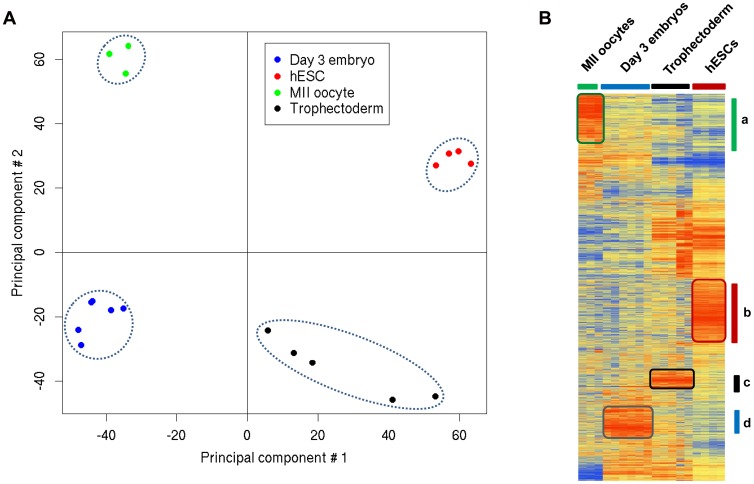
Gene expression patterns of day 3 human embryos, mature MII oocytes, TE cells and hESC cells. (A) PCA two-dimensional scatter plots represent the differential gene expression patterns of the different human samples. Each dot represents a sample and the color its origin: oocytes (green dots), day 3 embryos (blue dots), TE from day 5 embryos (black dots) and hESCs (red dots). Samples can be divided in four distinct areas based on their gene expression. (B) Average-link hierarchical clustering of 15,000 genes delineated four major gene clusters: (a) genes specifically detected in mature MII oocytes; (b) genes over-expressed in hESCs; (c) genes up-regulated in TE and (d) genes specifically over-expressed in day 3 embryos.

In order to determine the global gene expression variation in the different samples, we established the gene expression profile of mature MII oocytes (n = 3), day 3 single embryos (n = 6), TE samples from day 5 blastocysts (n = 5) and hESCs (n = 4) (to represent the ICM) by using high-density oligonucleotide Affymetrix HG-U133P microarray chips. A non-supervised analysis using the principal components analysis (PCA) showed that samples from the same group clustered together very tightly (Figure 1A), corroborating the robustness of the Affymetrix microarrays [12]. Moreover, a non-supervised hierarchical clustering analysis of the array data (based on 15,000 genes) clustered perfectly the different samples, confirming their very specific expression profiles (Figure 1B). Finally, a scatter plot analysis (Figure S1) showed that expression variations between mature MII oocytes and single day 3 embryos were high as illustrated by the dispersed scatter plots and the low correlation coefficient (0.51). Conversely, the differences in gene expression between day 3 embryos and TE or hESC samples were lower as indicated by the tighter scatter plots and the high correlation coefficients (0.60–0.76) (Figure S1). These results reveal dynamic transcriptome changes during the transition from mature oocyte to day 3 embryo and from day 3 embryo to blastocyst. These “dynamic patterns” are due to the large-scale degradation of human maternal transcripts and the activation of embryonic genes, as was also observed in the mouse [10], [13].

### Comparison of the Gene Expression Profiles of Day 3 Embryos and TE Cells Isolated from Day 5 Blastocysts

**Figure 2:**
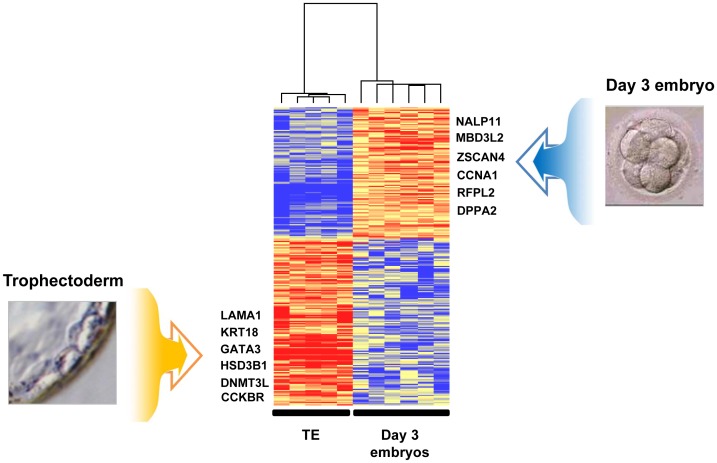
Day 3 embryo and TE molecular signatures: Heat map of the molecular signatures in six day 3 embryos and five TE samples. Each horizontal line represents a gene and each column represents a single sample. The color intensity indicates the level of gene expression (red for up-regulation and blue for down-regulation) “see also Table S1 and S2”.

**Figure 3:**
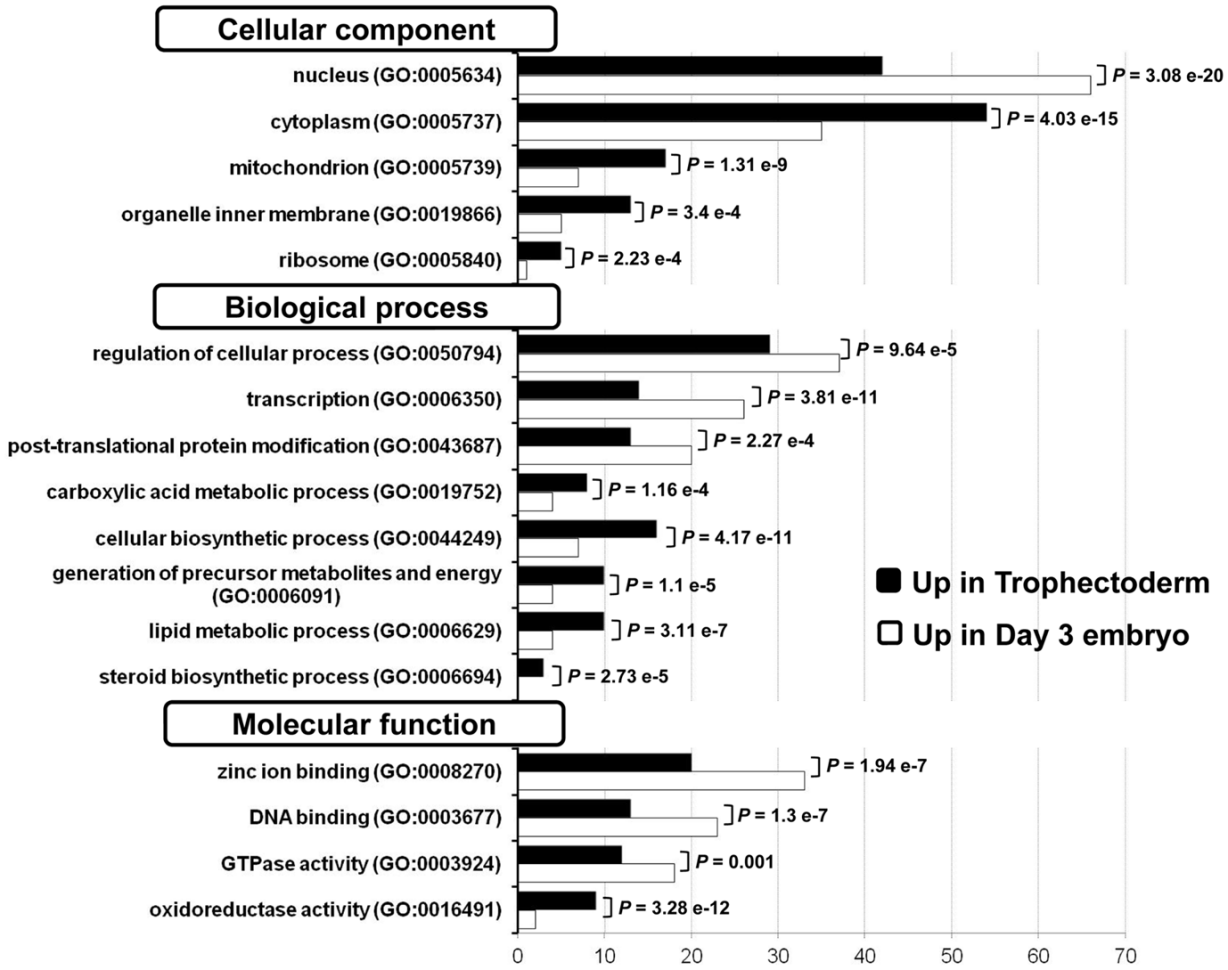
Gene Ontology (GO) annotations of the day 3 embryo and TE molecular signatures. We compared the GO annotations of genes specifically over-expressed in day 3 embryos and in TE cells by using the Babelomics web tool (http://babelomics.bioinfo.cipf.es/). Histograms show the percentage of genes with a specific GO annotation in day 3 embryos (white) or in TE samples (black). Only GO categories which differed significantly (*p* value <0.01) between the two groups are shown.

We then compared the expression profiles of day 3 embryos and TE cells, by using the significance analysis of microarrays (SAM) software with a 2-fold change cut-off and false discovery rate (FDR) <1%. We found that 2,196 transcripts were up-regulated in human TE cells (“TE molecular signature”) and 1,714 in day 3 embryos (“day 3 embryo molecular signature”) (Figure 2). The comprehensive lists of these signatures are presented in Tables S1 and S2 and the 100 genes with the highest fold change and significant statistical value (FDR = 0) for each signature are listed in Table 1 and 2. The “day 3 embryo molecular signature” included the Developmental Pluripotency Associated gene 5 (*DPPA5)*, members of the Ret finger protein-like gene family (*RFPL1, 2* and *3*), of the NLR family (*NALP4*, *5*, *9*, *11* and *13*), and of the melanoma antigen family (*MAGEA1*, *2*, *3*, *5*, *6* and *12*). Several maternal genes were found in this signature, such as members of the Zona Pellucida gene family (*ZP2*, *3* and *4*), *ZAR1*, *AURKC* and *FIGLA*, suggesting that they are still active in day 3 embryos. Several transcription factors were also significantly over-expressed in day 3 embryos, such as *TFB1M* and *TFB2M*, the transcriptional regulators *MBD3L2* and *ZSCAN4*, as well as metabolic genes such as Pyruvate Dehydrogenase Kinase 3 (*PDK3*) and Lactate Dehydrogenases (*LDHC*). The “TE molecular signature” comprised genes important for placental development (*PGF* and *TFAP2A*), cytoskeleton-associated genes (*Keratin 18* and *19*), and genes encoding S100 calcium binding proteins (*S100P*, *S100A6*, *10*, *13*, *14* and *16*), retinoid receptor-related testis-associated receptors (*NR2F2* and *NR2F6*) or the B receptor (*CCKBR*). Moreover, genes encoding extracellular matrix proteins, such as Laminins (*LAMA1*, *LAMA5* and *LAMC1*) and Integrins (*ITGB4* and *ITGB5*) were also up-regulated. Gene ontology (GO) annotations were used to explore the specific functional properties of the two molecular signatures (Figure 3). The day 3 embryo molecular signature was enriched in genes associated with localization in the “nucleus”, while genes associated with the “cytoplasm” localization were over-represented in the TE molecular signature. Concerning the “biological processes”, the day 3 embryo molecular signature was enriched in genes involved in the regulation of cellular processes, transcription and post-translational protein modifications. Conversely, in the TE molecular signature, genes connected with different metabolic and steroid biosynthetic processes were over-represented. The “molecular function” analysis showed that genes involved in oxido-reductase activity were significantly enriched in the TE signature (*p*<0.001), whereas genes related to “GTPase activity” and DNA binding were over-represented in the day 3 embryo signature. Finally, the expression pattern of 11 genes belonging to the TE (*GATA3*, *LAMA1*, *KRT18, HSD3B1*, *HSD17B1* and *DNMT3L*) or to the day 3 embryo molecular signature (*MBD3L2*, *CCNA1*, *BIK*, *RFPL2* and *FIGLA*) was confirmed by qRT-PCR analysis using specific primer pairs (Table S3). All qRT-PCR data were normalized to *GAPDH* to control for variations in mRNA recovery and RT efficiency (Figure S2).

**Table 1:** List of the 100 genes with the highest fold change in day 3 human embryos in comparison to TE samples.

Probesets	Gene Name	Gene Title	UniGene	ChromosomalLocation	Fold change	FDR (%)
1552531_a_at	NALP11	NLR family, pyrin domain containing 11	Hs.375039	chr19q13.42	1893	0
242334_at	NALP4	NLR family, pyrin domain containing 4	Hs.631533	chr19q13.42	892	0
214957_at	ACTL8	actin-like 8	Hs.2149	chr1p36.2-p35	755	0
1556096_s_at	UNC13C	unc-13 homolog C	Hs.443456	chr15q21.3	663	0
207443_at	NR2E1	nuclear receptor subfamily 2, group E, member 1	Hs.157688	chr6q21	625	0
1553619_a_at	TRIM43	tripartite motif-containing 43	Hs.589730	chr2q11.1	519	0
1552405_at	NALP5	NLR family, pyrin domain containing 5	Hs.356872	chr19q13.42	448	0
209160_at	AKR1C3	aldo-keto reductase family 1, member C3	Hs.78183	chr10p15-p14	431	0
1552456_a_at	MBD3L2	methyl-CpG binding domain protein 3-like 2	Hs.567667	chr19p13.2	394	0
1557085_at	TMEM122	placenta-specific 1-like	Hs.132310	chr11q12.1	387	0
39318_at	TCL1A	T-cell leukemia/lymphoma 1A	Hs.2484	chr14q32.1	342	0
234393_at	HDAC9	histone deacetylase 9	Hs.196054	chr7p21.1	315	0
1552912_a_at	IL23R	interleukin 23 receptor	Hs.200929	chr1p31.3	306	0
1552852_a_at	ZSCAN4	zinc finger and SCAN domain containing 4	Hs.469663	chr19q13.43	282	0
226117_at	TIFA	TRAF-interacting protein with a forkhead-associated domain	Hs.310640	chr4q25	275	0
222361_at	LOC643224	similar to tubulin, beta 8	Hs.551805	chr9q34.3	273	0
229105_at	GPR39	G protein-coupled receptor 39	Hs.432395	chr2q21-q22	255	0
225626_at	PAG1	phosphoprotein associated with glycosphingolipid microdomains 1	Hs.266175	chr8q21.13	230	0
1557544_at	C10orf80	chromosome 10 open reading frame 80	Hs.253576	chr10q25.1	209	0
210634_at	KLHL20	kelch-like 20 (Drosophila)	Hs.495035	chr1q24.1-q24.3	206	0
206343_s_at	NRG1	neuregulin 1	Hs.453951	chr8p21-p12	184	0
207213_s_at	USP2	ubiquitin specific peptidase 2	Hs.524085	chr11q23.3	182	0
1563120_at	Hs.623820	Homo sapiens, clone IMAGE:5528155, mRNA	Hs.630724		175	0
237131_at	LOC645469	hypothetical protein FLJ36032	Hs.297967	chr1q21.3	172	0
221630_s_at	DDX4	DEAD (Asp-Glu-Ala-Asp) box polypeptide 4	Hs.223581	chr5p15.2-p13.1	171	0
241550_at	DPPA5	developmental pluripotency associated 5	Hs.125331	chr6q13	167	0
217365_at	PRAMEF5	similar to PRAME family member 6		chr1p36.21	157	0
1570337_at	FIGLA	folliculogenesis specific basic helix-loop-helix	Hs.407636	chr2p13.3	157	0
206140_at	LHX2	LIM homeobox 2	Hs.445265	chr9q33-q34.1	154	0
229738_at	DNAH10	dynein, axonemal, heavy chain 10	Hs.622654	chr12q24.31	154	0
209785_s_at	PLA2G4C	phospholipase A2, group IVC (cytosolic, calcium-independent)	Hs.631562	chr19q13.3	149	0
237613_at	FOXR1	forkhead box R1	Hs.116679	chr11q23.3	147	0
236914_at	AW080028				137	0
210467_x_at	MAGEA12	melanoma antigen family A, 12		chrXq28	137	0
242128_at	OTX2	orthodenticle homolog 2 (Drosophila)	Hs.288655	chr14q21-q22	128	0
220535_at	FAM90A1	family with sequence similarity 90, member A1	Hs.196086	chr12p13.31	128	0
215048_at	SUHW2	suppressor of hairy wing homolog 2 (Drosophila)	Hs.43834	chr22q11.22	127	0
207934_at	RFPL1	ret finger protein-like 1	Hs.648249	chr22q12.2	127	0
209994_s_at	ABCB1	ATP-binding cassette, sub-family B (MDR/TAP), member 1	Hs.489033	chr7q21.1	125	0
207227_x_at	RFPL2	ret finger protein-like 2	Hs.157427	chr22q12.3	116	0
238218_at	LOC648473	hypothetical protein LOC648473			112	0
214603_at	MAGEA2	melanoma antigen family A, 2	Hs.169246	chrXq28	111	0
217590_s_at	TRPA1	transient receptor potential cation channel, subfamily A, member 1	Hs.137674	chr8q13	110	0
208312_s_at	PRAMEF1	PRAME family member 1	Hs.104991	chr1p36.21	109	0
223866_at	ARMC2	armadillo repeat containing 2		chr6q21	106	0
216001_at	LOC390999	PRAME family member 12	Hs.156406	chr1p36.21	106	0
213228_at	PDE8B	phosphodiesterase 8B	Hs.584830	chr5q14.1	104	0
1552807_a_at	SIGLEC10	sialic acid binding Ig-like lectin 10	Hs.284813	chr19q13.3	104	0
236205_at	Hs.13188	similar to ATP-binding cassette, sub-family C, member 6	Hs.13188	chr16p12.3	104	0
209942_x_at	MAGEA3	melanoma antigen family A, 3	Hs.417816	chrXq28	100	0
226271_at	GDAP1	ganglioside-induced differentiation-associated protein 1	Hs.168950	chr8q21.11	98	0
240031_at	AA994467	Baculoviral IAP repeat-containing 2	Hs.503704	chr11q22	98	0
209570_s_at	D4S234E	DNA segment on chromosome 4 (unique) 234 expressed sequence	Hs.518595	chr4p16.3	98	0
206207_at	CLC	Charcot-Leyden crystal protein	Hs.889	chr19q13.1	96	0
230626_at	TSPAN12	tetraspanin 12	Hs.16529	chr7q31.31	93	0
216034_at	SUHW1	suppressor of hairy wing homolog 1 (Drosophila)	Hs.178665	chr22q11.22	89	0
231756_at	ZP4	zona pellucida glycoprotein 4	Hs.136241	chr1q43	89	0
202388_at	RGS2	regulator of G-protein signalling 2, 24 kDa	Hs.78944	chr1q31	85	0
205747_at	CBLN1	cerebellin 1 precursor	Hs.458423	chr16q12.1	84	0
230753_at	LOC197135	hypothetical LOC197135	Hs.11594	chr15q21.1	83	0
236117_at	Hs.42747	Transcribed locus	Hs.42747		83	0
1556834_at	Hs.562766	CDNA clone IMAGE:5296106	Hs.562766		83	0
209278_s_at	TFPI2	tissue factor pathway inhibitor 2	Hs.438231	chr7q22	81	0
240318_at	AFMID	Arylformamidase	Hs.558614	chr17q25.3	80	0
1557257_at	BCL10	B-cell CLL/lymphoma 10	Hs.193516	chr1p22	80	0
236504_x_at	C6orf52	chromosome 6 open reading frame 52	Hs.61389	chr6p24.1	80	0
204438_at	MRC1	mannose receptor, C type 1	Hs.75182	chr10p12.33	80	0
1559108_at	VPS53	Vacuolar protein sorting 53 (S. cerevisiae)	Hs.461819	chr17p13.3	79	0
210180_s_at	SFRS10	splicing factor, arginine/serine-rich 10 (transformer 2 homolog, Drosophila)	Hs.533122	chr3q26.2-q27	77	0
214960_at	API5	apoptosis inhibitor 5	Hs.435771	chr11p12-q12	77	0
232692_at	TDRD6	tudor domain containing 6	Hs.40510	chr6p12.3	76	0
240731_at	LOC441316				76	0
230697_at	BBS5	Bardet-Biedl syndrome 5	Hs.233398	chr2q31.1	75	0
244206_at	ANUBL1	AN1, ubiquitin-like, homolog (Xenopus laevis)	Hs.89029	chr10q11.21	75	0
222921_s_at	HEY2	hairy/enhancer-of-split related with YRPW motif 2	Hs.144287	chr6q21	74	0
1557146_a_at	FLJ32252	hypothetical protein FLJ32252	Hs.250557	chr16p13.3	73	0
241382_at	PCP4L1	Purkinje cell protein 4 like 1	Hs.433150	chr1q23.3	73	0
226811_at	FAM46C	family with sequence similarity 46, member C	Hs.356216	chr1p12	73	0
44783_s_at	HEY1	hairy/enhancer-of-split related with YRPW motif 1	Hs.234434	chr8q21	73	0
239061_at	TPRXL	tetra-peptide repeat homeobox-like	Hs.638296	chr3p25.1	72	0
223562_at	PARVG	parvin, gamma	Hs.565777	chr22q13.2-q13	69	0
219352_at	HERC6	hect domain and RLD 6	Hs.529317	chr4q22.1	69	0
1553697_at	C1orf96	chromosome 1 open reading frame 96	Hs.585011	chr1q42.13	68	0
1568924_a_at	FLJ35834	hypothetical protein FLJ35834	Hs.159650	chr7q31.32	68	0
221314_at	GDF9	growth differentiation factor 9	Hs.25022	chr5q31.1	67	0
228737_at	C20orf100	chromosome 20 open reading frame 100	Hs.26608	chr20q13.12	66	0
240070_at	VSIG9	V-set and immunoglobulin domain containing 9	Hs.421750	chr3q13.31	66	0
231448_at	Tenr	testis nuclear RNA-binding protein	Hs.518957	chr4q27	65	0
214612_x_at	MAGEA6	melanoma antigen family A, 6	Hs.441113	chrXq28	64	0
206696_at	GPR143	G protein-coupled receptor 143	Hs.74124	chrXp22.3	62	0
205551_at	SV2B	synaptic vesicle glycoprotein 2B	Hs.592018	chr15q26.1	61	0
219686_at	STK32B	serine/threonine kinase 32B	Hs.133062	chr4p16.2-p16.1	61	0
230645_at	FRMD3	FERM domain containing 3	Hs.127535	chr9q21.32	60	0
1555396_s_at	LOC340602	similar to CG32656-PA	Hs.97053	chrXp11.22	59	0
237464_at	IMAA	SLC7A5 pseudogene	Hs.448808	chr16p12.2	58	0
212158_at	SDC2	syndecan 2 (heparan sulfate proteoglycan 1, cell surface-associated, fibroglycan)	Hs.1501	chr8q22-q23	57	0
220657_at	KLHL11	kelch-like 11 (Drosophila)	Hs.592134	chr17q21.2	57	0
223883_s_at	STK31	serine/threonine kinase 31	Hs.309767	chr7p15.3	57	0
222925_at	DCDC2	doublecortin domain containing 2	Hs.61345	chr6p22.1	56	0
210148_at	AF305239	homeodomain interacting protein kinase 3	Hs.201918	chr11p13	56	0

**Table 2:** List of the 100 genes with the highest fold change in TE samples in comparison to day 3 embryos.

Probesets	Gene Name	Gene Title	UniGene	Chromosomal Location	Fold Change	FDR (%)
205980_s_at	ARHGAP8	Rho GTPase activating protein 8/PRR5-ARHGAP8 fusion		chr22q13.31	514	0
218237_s_at	SLC38A1	solute carrier family 38, member 1	Hs.533770	chr12q13.11	469	0
201596_x_at	KRT18	keratin 18	Hs.406013	chr12q13	445	0
204515_at	HSD3B1	hydroxy-delta-5-steroid dehydrogenase, 3 beta- and steroid delta-isomerase 1	Hs.364941	chr1p13.1	383	0
227048_at	LAMA1	laminin, alpha 1	Hs.270364	chr18p11.31	372	0
34260_at	KIAA0683	TEL2, telomere maintenance 2, homolog (S. cerevisiae)	Hs.271044	chr16p13.3	361	0
224348_s_at	AF116709				341	0
223168_at	RHOU	ras homolog gene family, member U	Hs.647774	chr1q42.11-q42.3	310	0
204158_s_at	TCIRG1	T-cell, immune regulator 1, ATPase, H+ transporting, lysosomal V0 subunit A3	Hs.495985	chr11q13.2	283	0
212203_x_at	IFITM3	interferon induced transmembrane protein 3 (1–8 U)	Hs.374650	chr11p15.5	279	0
242705_x_at	Hs.592928	Full length insert cDNA clone YT86E01	Hs.592928		277	0
204351_at	S100P	S100 calcium binding protein P	Hs.2962	chr4p16	262	0
201650_at	KRT19	keratin 19		chr17q21.2	260	0
229125_at	ANKRD38	ankyrin repeat domain 38	Hs.283398	chr1p31.3	238	0
224646_x_at	H19	H19, imprinted maternally expressed untranslated mRNA	Hs.533566	chr11p15.5	208	0
221538_s_at	PLXNA1	plexin A1	Hs.432329	chr3q21.3	204	0
210381_s_at	CCKBR	cholecystokinin B receptor	Hs.203	chr11p15.4	196	0
217853_at	TNS3	tensin 3	Hs.520814	chr7p12.3	194	0
209771_x_at	CD24	CD24 molecule	Hs.644105	chr6q21	194	0
210201_x_at	BIN1	bridging integrator 1	Hs.193163	chr2q14	156	0
224579_at	Hs.592612	solute carrier family 38, member 1	Hs.533770	chr12q13.11	147	0
204720_s_at	DNAJC6	DnaJ (Hsp40) homolog, subfamily C, member 6	Hs.647643	chr1pter-q31.3	135	0
212444_at	Hs.632997	CDNA clone IMAGE:6025865	Hs.632997		135	0
203767_s_at	STS	steroid sulfatase (microsomal), arylsulfatase C, isozyme S	Hs.522578	chrXp22.32	135	0
215729_s_at	VGLL1	vestigial like 1 (Drosophila)	Hs.496843	chrXq26.3	134	0
227241_at	MUC15	mucin 15, cell surface associated	Hs.407152	chr11p14.3	133	0
204121_at	GADD45G	growth arrest and DNA-damage-inducible, gamma	Hs.9701	chr9q22.1-q22.2	125	0
212077_at	CALD1	caldesmon 1	Hs.490203	chr7q33	122	0
201787_at	FBLN1	fibulin 1	Hs.24601	chr22q13.31	121	0
202286_s_at	TACSTD2	tumor-associated calcium signal transducer 2	Hs.23582	chr1p32-p31	109	0
218571_s_at	CHMP4A	chromatin modifying protein 4A	Hs.279761	chr14q12	108	0
205829_at	HSD17B1	hydroxysteroid (17-beta) dehydrogenase 1	Hs.50727	chr17q11-q21	108	0
205093_at	PLEKHA6	pleckstrin homology domain containing, family A member 6	Hs.253146	chr1q32.1	105	0
209735_at	ABCG2	ATP-binding cassette, sub-family G (WHITE), member 2	Hs.480218	chr4q22	104	0
213050_at	COBL	cordon-bleu homolog (mouse)	Hs.99141	chr7p12.1	97	0
205081_at	CRIP1	cysteine-rich protein 1 (intestinal)	Hs.122006	chr14q32.33	93	0
209262_s_at	NR2F6	nuclear receptor subfamily 2, group F, member 6	Hs.466148	chr19p13.1	91	0
203438_at	STC2	stanniocalcin 2	Hs.233160	chr5q35.2	90	0
214285_at	FABP3	fatty acid binding protein 3, muscle and heart	Hs.584756	chr1p33-p32	89	0
209369_at	ANXA3	annexin A3	Hs.480042	chr4q13-q22	89	0
209723_at	SERPINB9	serpin peptidase inhibitor, clade B (ovalbumin), member 9	Hs.104879	chr6p25	88	0
209921_at	SLC7A11	solute carrier family 7, (cationic amino acid transporter, y+ system) member 11	Hs.390594	chr4q28-q32	87	0
216604_s_at	SLC7A8	solute carrier family 7 (cationic amino acid transporter, y+ system), member 8	Hs.632348	chr14q11.2	86	0
228949_at	GPR177	G protein-coupled receptor 177	Hs.647659	chr1p31.3	84	0
202007_at	NID1	nidogen 1	Hs.356624	chr1q43	84	0
209513_s_at	HSDL2	hydroxysteroid dehydrogenase like 2	Hs.59486	chr9q32	83	0
225520_at	MTHFD1L	methylenetetrahydrofolate dehydrogenase (NADP+ dependent) 1-like	Hs.591343	chr6q25.1	82	0
202023_at	EFNA1	ephrin-A1	Hs.516664	chr1q21-q22	81	0
205710_at	LRP2	low density lipoprotein-related protein 2	Hs.470538	chr2q24-q31	78	0
217764_s_at	RAB31	RAB31, member RAS oncogene family	Hs.99528	chr18p11.3	77	0
225516_at	SLC7A2	solute carrier family 7 (cationic amino acid transporter, y+ system), member 2	Hs.448520	chr8p22-p21.3	77	0
200832_s_at	SCD	stearoyl-CoA desaturase (delta-9-desaturase)	Hs.558396	chr10q23-q24	76	0
202418_at	YIF1A	Yip1 interacting factor homolog A (S. cerevisiae)	Hs.446445	chr11q13	74	0
200872_at	S100A10	S100 calcium binding protein A10	Hs.143873	chr1q21	74	0
209603_at	GATA3	GATA binding protein 3	Hs.524134	chr10p15	73	0
1555832_s_at	KLF6	Kruppel-like factor 6	Hs.4055	chr10p15	73	0
202737_s_at	LSM4	LSM4 homolog, U6 small nuclear RNA associated (S. cerevisiae)	Hs.515255	chr19p13.11	71	0
226604_at	TMTC3	transmembrane and tetratricopeptide repeat containing 3	Hs.331268	chr12q21.32	71	0
220139_at	DNMT3L	DNA (cytosine-5-)-methyltransferase 3-like	Hs.592165	chr21q22.3	70	0
206269_at	GCM1	glial cells missing homolog 1 (Drosophila)	Hs.28346	chr6p21-p12	69	0
203743_s_at	TDG	thymine-DNA glycosylase	Hs.584809	chr12q24.1	69	0
219010_at	C1orf106	chromosome 1 open reading frame 106	Hs.518997	chr1q32.1	69	0
225021_at	ZNF532	zinc finger protein 532	Hs.529023	chr18q21.32	69	0
205524_s_at	HAPLN1	hyaluronan and proteoglycan link protein 1	Hs.591758	chr5q14.3	68	0
206548_at	FLJ23556	hypothetical protein FLJ23556		chr10q26.11	66	0
202800_at	SLC1A3	solute carrier family 1 (glial high affinity glutamate transporter), member 3	Hs.481918	chr5p13	65	0
229699_at	Hs.61558	CDNA FLJ45384 fis, clone BRHIP3021987	Hs.61558		65	0
229830_at	Hs.376032	Transcribed locus	Hs.535898		65	0
202308_at	SREBF1	sterol regulatory element binding transcription factor 1	Hs.592123	chr17p11.2	64	0
203219_s_at	APRT	adenine phosphoribosyltransferase	Hs.28914	chr16q24	64	0
225078_at	EMP2	epithelial membrane protein 2	Hs.531561	chr16p13.2	64	0
218180_s_at	EPS8L2	EPS8-like 2	Hs.55016	chr11p15.5	63	0
201440_at	DDX23	DEAD (Asp-Glu-Ala-Asp) box polypeptide 23	Hs.130098	chr12q13.12	62	0
201236_s_at	BTG2	BTG family, member 2	Hs.519162	chr1q32	62	0
218721_s_at	C1orf27	chromosome 1 open reading frame 27	Hs.371210	chr1q25	61	0
223062_s_at	PSAT1	phosphoserine aminotransferase 1	Hs.494261	chr9q21.2	61	0
201613_s_at	AP1G2	adaptor-related protein complex 1, gamma 2 subunit	Hs.343244	chr14q11.2	60	0
211986_at	AHNAK	AHNAK nucleoprotein (desmoyokin)	Hs.502756	chr11q12.2	60	0
223449_at	SEMA6A	sema domain, transmembrane domain (TM), and cytoplasmic domain, (semaphorin) 6A	Hs.156967	chr5q23.1	60	0
1567107_s_at	TPM3	tropomyosin 4	Hs.631618	chr19p13.1	58	0
208659_at	CLIC1	chloride intracellular channel 1	Hs.414565	chr6p22.1-p21.2	57	0
202546_at	VAMP8	vesicle-associated membrane protein 8 (endobrevin)	Hs.534373	chr2p12-p11.2	57	0
227042_at	LOC150223	hypothetical protein LOC150223	Hs.355952	chr22q11.21	57	0
202625_at	LYN	v-yes-1 Yamaguchi sarcoma viral related oncogene homolog	Hs.651186	chr8q13	56	0
235436_at	BE503800				55	0
223839_s_at	Hs.597496	PRO1933	Hs.597496		55	0
202830_s_at	SLC37A4	solute carrier family 37 (glycerol-6-phosphate transporter), member 4	Hs.132760	chr11q23.3	54	0
228834_at	TOB1	transducer of ERBB2, 1	Hs.649528	chr17q21	54	0
210589_s_at	GBA	glucosidase, beta; acid (includes glucosylceramidase)	Hs.282997	chr1q21	53	0
208683_at	CAPN2	calpain 2, (m/II) large subunit	Hs.350899	chr1q41-q42	53	0
201428_at	CLDN4	claudin 4	Hs.647036	chr7q11.23	52	0
217775_s_at	RDH11	retinol dehydrogenase 11 (all-trans/9-cis/11-cis)	Hs.226007	chr14q24.1	51	0
208613_s_at	FLNB	filamin B, beta (actin binding protein 278)	Hs.476448	chr3p14.3	49	0
230204_at	AU144114				49	0
209710_at	GATA2	GATA binding protein 2	Hs.367725	chr3q21.3	48	0
215464_s_at	TAX1BP3	Tax1 (human T-cell leukemia virus type I) binding protein 3	Hs.12956	chr17p13	47	0
1559266_s_at	FLJ45187	hypothetical protein LOC387640	Hs.350848	chr10p12.31	47	0
202090_s_at	UQCR	ubiquinol-cytochrome c reductase, 6.4 kDa subunit	Hs.534521	chr19p13.3	47	0
209652_s_at	PGF	placental growth factor, vascular endothelial growth factor-related protein	Hs.252820	chr14q24-q31	47	0
232164_s_at	EPPK1	epiplakin 1	Hs.200412	chr8q24.3	47	0

### Expression of Genes Encoding Proteins which Play a Role in Apoptosis in Day 3 Embryos and TE Samples

**Figure 4:**
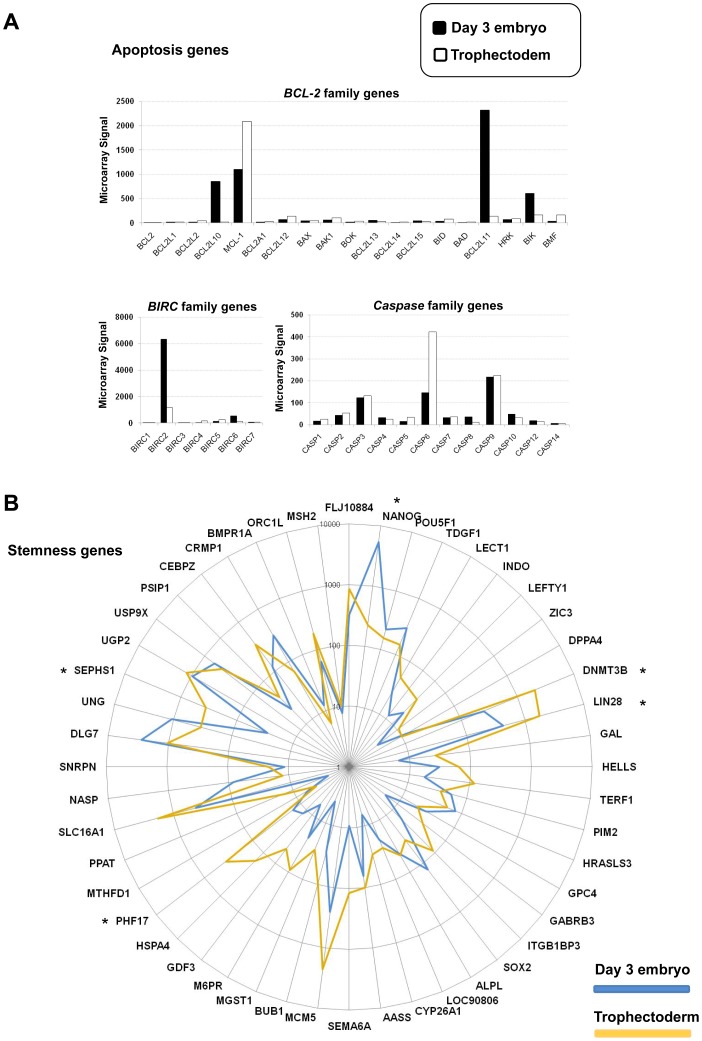
Differential expression of apoptosis and stemness-related genes in day 3 embryos and TE samples. (A) Histograms show the microarray signal values for apoptosis-related genes in day 3 embryos (black) and TE samples (white). (B) The mean expression level of 48 stemness genes in six day 3 embryos and five TE samples was plotted on a logarithmic scale in a radar graph. Asterisks indicate a statistically significant difference (*P*<0.05) between TE and day 3 embryos (Mann-Whitney test).

We then investigated the expression of genes coding for proteins linked to the extrinsic and intrinsic apoptosis pathways in day 3 embryos and TE cells. The expression of genes of the *TNF* ligand and receptor family was not different in day 3 embryos and TE cells. Conversely, several genes belonging to the *BCL-2*, *BIRC* and *Caspase* families appeared to be differentially expressed in the two groups (Figure 4A). Specifically, the *BCL-2* family members *BCL2L10* (×37, FDR <0.0001), *BCL2L11* (×16, FDR <0.001), and *BIK* (×3.7, FDR <0.001*)*, the expression of which was validated by qRT-PCR (Figure 3), and the *BIRC* family member *BIRC2* (×4, FDR <0.001) were up-regulated in day 3 embryos. *Caspase 6* (×3, FDR <0.001) was over-expressed in TE cells. *MCL-1*, a gene that belongs to the *BCL2* family and promotes cell survival, was strongly expressed in both day 3 embryos and TE samples.

### Evaluation of DNA Repair Regulation in Day 3 Embryos and TE Samples

The microarrays data were also used to investigate the expression of a comprehensive list of DNA repair genes [14] in day 3 embryos and TE samples (Tables S1 and S2). Of the 123 DNA damage repair genes investigated, five [*UNG*, *RFC1*, *UNG2* (now named *CCNO*), *PCNA*, *MSH2*] were up-regulated in day 3 embryos and eleven [*BRCA1*, *TDG*, *FANCG*, *FEN1*, *XRCC5*, *XRCC6*, *XPC*, *MUTYH*, *XPA*, *SMUG1*, *POLD2*] in TE cells. We then analyzed the functional relationship between the DNA damage repair genes that were differentially expressed in TE samples and day 3 embryos using the Ingenuity Pathway Analysis (IPA) software. In both cases, all the DNA repair genes displayed a documented functional interaction with each other, forming a tightly connected network (Figure S3).

### Stemness Genes and Transcriptional Regulatory Networks Identified in Day 3 Embryos and TE Cells

**Figure 5:**
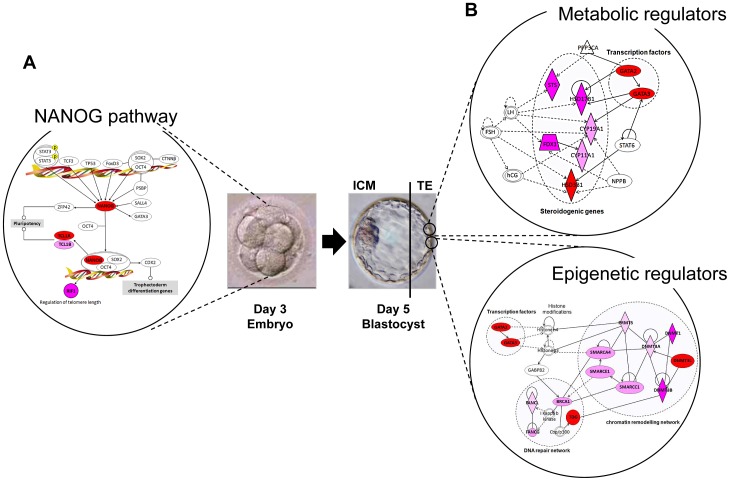
Up-regulated genes that are related to the *NANOG* pathway, or to metabolic and epigenetic functions in day 3 human embryos and TE samples. (A) The interaction network was generated with the Ingenuity software and shows that many genes from the *NANOG* pathway are up-regulated (red) in day 3 embryos. (B) Top-ranked functional networks in which are involved transcription factors (*GATA2* and *GATA3*), or genes that regulate steroidogenesis (including *HSD1B3*), DNA repair (*TDG* and *BRCA1*) or epigenetic modifications (including *DNMT3L)* and that are up-regulated in TE samples. The color intensity indicates their degree of up-regulation. Uncolored genes were identified as not differentially expressed by our analysis, but were, nevertheless, integrated into the computationally generated networks on the basis of the evidence stored in the IPA knowledge memory indicating a relevance to this network. In each network, nodes indicate genes, a plain line indicates direct interaction, a dashed line indicates indirect interaction; a line without arrowhead indicates binding only; a line with an arrowhead indicates “acts on”.

We then performed a stemness gene enrichment analysis using a previously published dataset from hESCs, in which we defined a consensus hESC stemness gene list (n = 48 genes) [7]. The key stemness factors *NANOG*, *POU5F1* (*OCT3/4*) and *SOX2* [15] were enriched in day 3 human embryos, whereas *DNMT3B*, *LIN28*, *PHF17*, *SEPHS1* were over-represented in TE cells. Conversely, other genes, such as *UGP2* and *PIM2,* were enriched in both day 3 embryos and TE samples (Figure 4B). Bioinformatic gene pathway analysis (Ingenuity software) of the day 3 embryo molecular signature showed that many genes of the *NANOG* signaling pathway, including *NANOG* (Figure 5), were up-regulated in day 3 human embryos, thus confirming the role of *NANOG* in the maintenance of pluripotency [16]. The “TE molecular signature” included transcription factors such as *GCM1*, which is induced by Transforming Growth Factor-β (*TGF-β*) [17], and Bone Morphogenic Protein 4 (*BMP4*) that induces the differentiation of pluripotent stem cells to trophoblast cells [18], [19]. Other components of the *TGF-β* signaling cascade, such as Transforming Growth Factor Beta Receptor III (*TGFBRIII*), were also included in the “TE molecular signature”.

### Dynamic Expression of Epigenetic and Metabolic Regulators During Trophoblast Development

Since specification of the TE lineage during blastocyst formation involves initiation of differentiation, it is likely that epigenetic regulators may have an important role in this first developmental decision. The majority of the epigenetic regulators that were up-regulated in TE cells are associated with a repressive epigenetic status (Figure 5). Specifically, the expression of the DNA methyl transferases (DNMT) *DNMT3A*, *DNMT3B* and *DNMT1* increased between 2- and 13-fold in TE cells in comparison to day 3 embryos. *DNMT3L* expression was 70-fold higher in TE samples than in day 3 embryos. Similarly, several transcripts coding for proteins involved in chromatin remodeling and histone modification (*SMARCA4*, *SMARCC1* and *SMARCE1*) were up-regulated between 2- and 7-fold in TE cells. Conversely, many histone deacetylases (*HDAC9*and *HDAC2*) and histone acetyltransferases (*HAT1*, *SETD8*, *RNF20*, *TAF1*, *STK17B*, *31*, *32B* and *35*) were down-regulated in TE cells in comparison to day 3 embryos. Another feature of the TE molecular signature was the up-regulation of several metabolic genes. Specifically, genes that are involved in estrogen biosynthesis (*CYP11A1* x35, *CYP19A1* x14) and lipid metabolism (*PTGES* x20) were strongly up-regulated in TE cells. One of the most striking observations was the high expression of genes that are involved in steroidogenesis (*HSD3B1* ×383, *STS* ×135, *HSD17B1* ×108, *FDX1* ×14 and *SRA1* ×6).

### Intersection with the Transcriptomes of Mature MII Oocytes and hESCs

In an effort to link the genes involved in the day 3 embryo-TE transition with early embryonic development, we further investigated differences and similarities in the gene expression patterns of MII oocytes, day 3 embryos, TE cells and hESCs samples (comprehensive list in Table S4). The genes that were found to be up-regulated in day 3 embryos (Table S1) and TE cells (Table S2) were individually compared to those up-regulated in MII oocytes and hESCs using Venn diagrams (Figure S4). Only 36 genes were common to both the TE and the MII oocyte signatures. On the other hand, day 3 embryos and MII oocytes shared a set of 511 genes, among which many are associated with oogenesis, such as *DAZL*, *GDF9* and *FIGLA*. Finally, 1263 genes were common to both TE and hESC profiles, whereas only 124 genes were shared by day 3 embryos and the hESCs. Genes that were up-regulated in both TE and hESC samples were associated with cell death and proliferation (*BAG6*, *CASP2* and *ANXA3*), metabolism (*GCDH* and *HPGD*) and WNT signaling (*FZD5*, *AXIN1* and *TCF3*). Genes that were up-regulated in both day 3 embryos and hESCs (124 genes) are involved in the maintenance of pluripotency and tissue development, such as *NANOG*. Among the genes specifically up-regulated in TE samples (644 genes), key genes related to epigenetic and metabolic pathways, such as *DNMT3L*, *HSD3B1* and *HSD17B1*, were observed.

## Discussion

Here, we compared the transcriptomes of day 3 human embryos and TE cells from day 5 human blastocysts to identify transcripts that are differentially expressed during the embryo-to-TE transition and the specification of the TE cell lineage. Many of the genes that were up-regulated in TE cells are already known to be associated with human TE differentiation [20], [21]. For instance, we confirmed that *GATA3* and *KRT18*, two trophoblast-determining genes, are enriched in TE from human blastocysts [22]. Moreover, the “TE molecular signature” included also unexpected genes, the TE-specificity of which has been overlooked. For instance, *CCKBR* activates signaling pathways involved in cell proliferation or migration [23], [24] and stimulates the expression of *β*1-Integrin *in vitro* [25]. A number of cell adhesion genes that might be implicated in the embryo attachment to the endometrium were also up-regulated in TE cells, including members of the Integrin family (*ITGB5)* and genes related to extracellular matrix remodeling, such as Laminins (*LAMA1* and *LAMC1*). In humans, active steroid hormones, including progesterone that is secreted by mouse TE cells [26], are essential for implantation and maintenance of pregnancy. Our analysis reveals that *HSD3B1*, *HSD17B1* and *FDX1*, which encode enzymes involved in the metabolism of cholesterol, were specifically up-regulated in TE cells in comparison to day 3 embryos (Figure S5). Moreover, *PTGES* (Prostaglandin E synthase) as well as *CYP11A1* and *CYP19A1* (estrogen synthesis) were also up-regulated in TE cells, suggesting a central role of these steroidogenic enzymes in TE steroid biosynthesis and metabolism. Thus, the TE joins the group of tissues with “steroidogenic” activity, such as brain, heart, gonads, endometrium and placenta [27], [28]. It is now important to compare the steroidogenic gene expression profiles in TE cells isolated from good and bad quality blastocysts to fully correlate specific transcriptional events with efficient TE development.

Among the models used to study trophoblast development, hESCs have emerged as a useful tool to examine the emergence and differentiation of TE cells. Particularly, the transcriptomic analysis of TE cells derived from hESCs has provided new insights into the signaling pathways and the molecular mechanisms underlying early trophoblast development. Recently, by using a microarray approach, Marchand and colleagues investigated gene expression during differentiation of hESCs into the trophoblast lineage upon addition of Bone Morphogenetic Protein 4 (BMP4) for 10 days and identified 670 genes that were up-regulated from day 0 to day 10 [29]. By intersecting these genes with those we found to be up-regulated in TE cells isolated from day 5 embryos, we found 104 common genes (see Table S5) among which there were not only trophoblast markers (for instance, *GATA3* and *KRT19*), but also many genes implicated in lipid metabolism and estrogen biosynthesis (i.e., *CYP19A1*, *CYP11A1*, *HSD17B1*, *HSD3B1*, *PTGES*, *STS*, *HPGD*, *SLCO2A1*, *HMOX1*, *ABCG2*, *ASAH1* and *SMPD1*). This finding validates the importance of metabolic genes during TE specification. Aghajanova et al. [30] compared the transcriptome of embryo-derived TE cells with that of hESC-derived TE cells and found that most of the shared genes were involved in the development of receptive endometrium during implantation. Suzuki et al. [31] used human embryonic carcinoma cells (G3), which can differentiate into TE cells, as an experimental model to investigate the molecular mechanism of trophoectoderm differentiation. Thus, comparative studies using human TE and hESC or G3 cells are relevant to better understand the molecular basis of cell fate decisions and to develop models of human TE development.

The “day 3 embryo molecular signature” was enriched in genes from the *NLRP* (named *NALP*) family which might play a role in early embryo development [32], [33]. Indeed, *NLRP5*, *NLRP8* and *NLRP9* are expressed in bovine and human pre-implantation embryos [32], [34] and, in pregnant *NLRP*5 null female mice, embryo development is arrested at the two-cell stage [35]. Remarkably, many genes of the day 3 signature belong to the Melanoma Antigen family and the Ret finger protein-like family. Most of their functions remain largely unknown, but some of them are thought to regulate, respectively, placenta and early embryo development [36], [37]. Mouse data suggest that two other day 3 embryo-specific genes (*MBD3L2* and *ZSCAN4)* might regulate early embryo development. In mouse embryos, *MBD3L2* expression coincides with EGA [38] and *ZSCAN4* (zinc finger and SCAN domain containing 4) is important for the progression from the 2-cell to 4-cell stage [39]. *ZSCAN4* plays also a key role in defying cellular senescence and maintaining a normal karyotype during propagation of embryonic stem cells in culture [40]. Additionally, the expression levels of *DPPA5*, *DPPA2* and the stemness factor *NANOG* were much higher in day 3 embryos than in TE samples. The reciprocal pattern of expression of *Nanog* and the transcription factors *Gata6* and *Cdx2* in the mouse morula suggests that *Nanog* might determine ICM pluripotency by repressing *Gata6* and *Cdx2*, which are implicated in the extra-embryonic lineage specification [41].

Our transcriptome analysis also shows that the TE molecular signature includes many genes that are annotated as “membrane”, demonstrating a strong bias towards genes involved in cell-to-cell communication processes. Conversely, genes specifically expressed by day 3 embryos are largely “nuclear”. Additionally, we categorized the genes that were up-regulated during the MII-day 3 transition according to their molecular and cellular function using the GO annotations and found that they were mainly associated with nuclear localization. This is in line with previously published data showing that proteins produced by the most up-regulated genes during the MII-day 2 embryo transition are mainly localized in the nucleus [11] and that hESC-specific genes are significantly depleted in extracellular signaling components [7]. One assumption that can be inferred from these findings is that the determinants of the MII-embryo transition and pluripotency may be regulated by intrinsic factors.

Apoptotic cell death has been observed in human and other mammalian pre-implantation embryos [42]. The expression profile of apoptosis-related genes in day 3 embryos suggests that the balance between anti- (*BCL2L10* and *BIRC2*) and pro-apoptotic factors (*BCL2L11* and *BIK*) might be critical at this stage of development. As the onset of EGA occurs at day 3 post-fertilization in humans, embryos that fail to accurately activate their genome might be committed to death by default. In contrast to mouse blastocysts where apoptosis occurs predominantly in ICM cells [43], apoptotic nuclei have been detected in both ICM and TE cells in human blastocysts [44]. Accordingly, we show that some molecular actors of apoptosis signaling are up-regulated in human TE cells (i.e. *Caspase 6*, *MCL-1*).

The expression of some DNA repair genes has been detected in mammalian embryos at different stages of development [45]. Our data show that two “DNA damage sensor” genes (*RFC1* and *PCNA1*) and two “base excision repair” genes (*UNG* and *UNG2* (now named as *CCNO*)) are up-regulated in human day 3 embryos, in line with previous works [46], and three “Double strand break repair” genes (*BRCA1*, *XRCC5* and *XRCC6*) are over-expressed in TE cells. In homozygous *Brca1^5–6^* mouse mutants, in which exons 5 and 6 of *Brca1* were deleted, the development of the extra-embryonic region was abnormal and diploid trophoblast cells were absent [47]. This may indicate that the “Double strand break repair” activity may be important for TE specification.

Epigenetic mechanisms, including DNA methylation, are key elements for controlling gene expression during the embryo-TE transition. In mouse blastocysts, DNA methyltransferase expression is restricted to the ICM, in which nuclei are highly methylated [48], whereas in human and bovine blastocysts, DNA methylation is higher in TE than ICM cells [49]. Here we report a strong expression of DNA (cytosine-5) methyltransferases (*DNMT3A*, *DNMT3B*, *DNMT1* and *DNMT3L*) in human TE cells (Figure 5). *DNMT3A* and *DNMT3B* are de novo enzymes that establish methylation patterns. *DNMT1* is a maintenance enzyme involved in preserving already acquired methylation patterns. *DNMT3L* lacks a catalytic domain, but can interact with the de novo enzymes [50], stimulating their activity [51]. Comparison with other samples including MII oocytes and hESCs suggests that *DNMT3L* is specifically up-regulated in TE cells (Figure S4). However, DNA methylation levels have been described to be globally low in extra-embryonic tissues in both mouse and human embryos [52], [53]. In these tissues, DNA (cytosine-5) methyltransferases enzymes are expressed only transiently and do not contribute to adult tissues maintenance, thus long-term epigenetic reprogramming may not be critical for extra-embryonic tissues. Moreover, the high expression of different epigenetic regulators in human TE cells could be a consequence of *in vitro* embryo culture. Studies in animal models have demonstrated that under certain *in vitro* culture conditions, DNA methylation profiles can be altered [54]. In another hand, the association between *in vitro* culture conditions during assisted reproduction and increased risk of some epigenetic disorders has been reported, clearly indicating that epigenetic deregulation must be considered when examining in vitro fertilized embryos. Our findings suggest that epigenetic modifiers cooperate with transcription factors and DNA repair genes to regulate the whole gene expression profile in TE cells (Figure 5). Disruption of this epigenetic regulatory circuit might lead to alterations of the normal physiological functions. Therefore, a comprehensive elucidation of this regulatory network would be highly beneficial for understanding TE anomalies and for improving assisted reproduction procedures. Moreover, a better knowledge on the TE-specific genes and the transcriptional networks operative in TE cells and day 3 embryos might led to the identification of new biomarkers that might be used as diagnostic tools to monitor the health, viability and competence of embryos in assisted reproduction programs.

### Limitations

As the day 3 embryos and the day 5 embryos used to isolate TE cells were donated from infertile women who underwent IVF treatments, the gene expression profiles could be have been influenced by the controlled ovarian stimulation (COS) carried out during IVF and thus they might not completely reflect the physiological situation under natural cycles. Moreover, due to the bioethics law that regulates the research on human embryos in France, the number of embryos donated for research is smaller. In view of these limitations, we optimized our technique to obtain transcriptome data for each single embryo and trophectoderm sample, respectively.

## Materials and Methods

### Specimen Collection and Processing

Human day 3 (post-fertilization) embryos and day 5 blastocysts were donated for research by infertile couples undergoing IVF treatment. All patients signed informed consent forms and the protocol for collecting human embryos and TE was approved by the Ethical Committee of the French National Agency of Biomedicine.

#### Day 3 embryos

9 embryos from 6 different couples were used for microarray analyses (n = 6) and qRT-PCR validation (n = 3). Day 3 embryos were all 6–8 cells with <20% fragmentation. Each embryo was individually transferred in a tube containing extraction buffer and frozen at −80°C for subsequent RNA extraction.

#### Trophectoderm biopsy

8 day 5 blastocysts were used for TE isolation for microarray analyses (n = 5) and qRT–PCR validation (n = 3). Blastocysts were fully expanded with a well-defined ICM and TE was scored according to Gardner [55]. After removal of the zona pellucida, TE was mechanically dissected from ICM. All TE samples were immediately transferred in tubes containing RLT lysis buffer and frozen at −80°C.

#### Mature MII oocytes and hESCs

After informed consent, unfertilized MII oocytes were collected 24 or 48 hours post-insemination as previously described [56]. Briefly, three pools of 16 MII oocytes (6 patients), 21 MII oocytes (8 patients) and 24 MII oocytes (8 patients) provided from couples referred to our center for conventional IVF for tubal infertility or for ICSI for male infertility were used for microarray analyses and qRT-PCR validation. The three hESC lines (HD83, HD90 and HD129) were derived by our group. Briefly, derivation of these lines was carried out using mechanical extraction of the inner cell mass [57]. The culture medium used for hESC derivation and culture consisted of 80% KO-DMEM, 20% Knockout serum replacement (KO-SR), 0.1 mM non-essential amino acids, 2 mM L-Glutamine, 0.5 mM β-mercaptoethanol and 10 ng/mL of bFGF. Passaging was performed mechanically by cutting the colony using a #15 scalpel under microscope. Mitotically inactivated (by irradiation) human foreskin fibroblasts (HFF) were used as feeder cells. HFFs were cultured in 85% DMEM, 15% FBS. HD83, HD90 and HD129 hESC lines were used for microarray analyses and HD90, HD129 and HS181 (imported from the Karolinska Institute (Stockholm, Sweden)) hESC lines were used for qRT–PCR validation.

#### RNA extraction

The RNeasy Micro kit (Qiagen) was used to isolate total RNA from TE samples and the Picopure RNA isolation kit (Arcturus Reagents/Molecular Devices, KIT0204, USA) for day 3 embryos, according to the manufacturers’ recommended protocols. The quantity and purity of the total RNAs were determined by using a NanoDrop® ND-1000 spectrophotometer (NanoDrop ND-Thermo Fisher Scientific, Wilmington, DE, USA) and their integrity by using the Agilent 2100 Bioanalyzer (Agilent Technologies, Palo Alto, CA, http://www.agilent.com).

### Complementary RNA (cRNA) Preparation and Microarray Processing

Total day 3 embryo RNA samples (from 450 pg to 855 pg) were subjected to three rounds of linear amplification and total TE RNA samples (between 50 and 100 ng) were twice amplified to generate suitable quantity of labeled cRNA for hybridization to HG-U133 plus 2.0 GeneChip arrays (Affymetrix, Santa Clara, CA, USA) as described in [9] and following the standard Affymetrix instructions. Briefly, RNA was amplified from individual human embryos using the RiboAmp® HS Kit according to manufacturer’s instructions (Arcturus Bioscience). During the first strand synthesis reaction, cDNA that incorporates a T7 promoter sequence is produced. This cDNA was then used as a template for the in vitro-transcription reaction driven by the T7 promoter to synthesize antisense RNA (aRNA), which was used as input for the second round of amplification. cRNA was then transcribed into cDNA and the T7 promoter was used to drive the second round of in vitro transcription. The double-stranded cDNA was then subjected to three rounds of linear amplification. The amplified aRNA was labeled with biotin using the Turbo Labeling Kit (Arctutus) and fragmented. Finally, fifteen micrograms of each labeled sample were hybridized to the HG-U133plus2 GeneChip array (Affymetrix). The microarray data were obtained in agreement with the Minimal Information about Microarray Experiment (MIAME) recommendations [58]. All data are accessible at the US National Center for Biotechnology Information, Gene Expression Omnibus (GEO) repository http://www.ncbi.nlm.nih.gov/geo through the provisional accession series number GSE33025.

### Data Processing and Visualization

After image processing using the Affymetrix Microarray Suite 5.0, the cell files were analyzed using the Affymetrix Expression Console software and normalized with the MAS5 algorithm by scaling each array to a target value of 100 using the global scaling method to obtain an intensity value signal for each probe set. Gene annotation was performed using NetAffx (http://www.affymetrix.com; March 2009). Genes with significant differential expression profiles were identified using the two-class Significance Analysis of Microarray (SAM) algorithm (http://www-stat.stanford.edu/~tibs/SAM/) with the Wilcoxon test and sample label permutation (n = 300). Briefly, the algorithm assigns a score to each gene based on differences in expression between conditions relative to the standard deviation of repeated measurements. The false discovery rate (FDR) is determined using permutations of the repeated measurements to estimate the percentage of genes identified by chance. The algorithm was applied to each dataset separately using FDR<1%. Subsequently, only the genes marked as significantly up-regulated or down-regulated were considered as differentially expressed in TE or embryos compared with the other samples. For hierarchical clustering, data were log-transformed, median-centered and processed with the CLUSTER and TREEVIEW software packages [59]. To cluster the samples according to the similarity of their gene expression patterns, we performed an unsupervised principal component analysis (PCA) with the RAGE bioinformatics platform [http://rage.montp.inserm.fr/] to project samples onto three-dimensional spaces that were further visualized to see the constellation of all samples using all the detected genes. The expression of selected genes in the panel of samples that includes germinal, stem cells and adult tissues, were retrieved through our “Amazonia!” database (http://amazonia.montp.inserm.fr/). The Ingenuity Pathways Analysis (IPA) system (Ingenuity Systems, Redwood City, CA, USA) was used to identify networks related to the genes that were differentially expressed in day 3 embryos and TE samples.

### Gene Ontologies (GO) Classification

Gene Ontology (GO) annotation analysis was carried out using the Fatigo+ tool http://babelomics.bioinfo.cipf.es [60] to identify biologically relevant themes among the genes that were differentially expressed in day 3 embryos and TE cells. Briefly, Fatigo+ performs a functional enrichment analysis by comparing two lists of genes by means of the Fisher’s Exact Test. Gene modules used in the test are defined in different ways that include functional criteria (GO, KEGG, Biocarta, etc.). Also user-defined gene modules can be imported and used for functional enrichment.

### Validation of Microarray Data by Quantitative RT–PCR Amplification

Gene expression profiles derived from microarray analyses were confirmed quantitatively by real-time qRT-PCR analysis using RNAs from three TE samples, three day 3 embryos, three MII oocytes and three hESC samples. The primer sequences are shown in Table S3. Briefly, cDNA was reverse transcribed following the manufacturer’s instructions using 500 ng of total RNA in a 20 µl reaction that contained Superscript II (Invitrogen), oligo dT primer, dNTP mixture, MgCl2 and RNase inhibitor. Aliquots of cDNA (1/25 of the RT reaction) were diluted in 50 µl reaction volume. Q-PCR was performed using a LightCycler 480 apparatus with the LC480 SYBR Green I Master kit (Roche Diagnostics, Mannheim, Germany) containing 2 µl cDNA and 0.6 mMol primers in a total volume of 10 µl. After 10 min of activation at 95°C, cycling conditions were 10 s at 95°C, 30 s at 63°C and 1 s at 72°C for 50 cycles. Gene expression levels were normalized to *GAPDH* using the following formula 100/2^ΔΔCt^ where ΔΔCt = ΔCt unknown - ΔCt positive control. Statistical comparisons were carried out using the Student’s *t* test and the SPSS software. *P* values less than or equal to 0.05 were considered significant.

## Supporting Information

Figure S1 **Scatter plots showing the comparative distribution of transcripts in mature MII oocytes, day 3 embryos, TE and hESC samples.** Each sample was plotted against all the other samples to visualize expression variations. The blue areas highlight a greater than two-fold gene expression difference (up-regulated) between the X-axis and Y-axis samples. The orange areas indicate a greater than two-fold gene expression difference (down-regulated) between the X-axis and Y-axis samples. The yellow areas highlight a 0.5- to 2-fold gene expression difference between the X-axis and Y-axis samples. For each couple of samples, the Pearson’s correlation coefficient was computed (*r*).
(TIF)

Figure S2 **Quantitative RT–PCR validation of the microarray results:** All qRT–PCR results were normalized to the expression of *GAPDH* in each sample and are the mean ± SEM of individual day 3 embryos (n = 3), TE (n = 3), pooled MII oocyte (n = 3) and hESC (n = 3) samples analyzed in duplicate. *P<0.05 was considered significant
(TIF)

Figure S3 **IPA results showing the network of DNA repair genes that are up-regulated in TE samples from day 5 human blastocysts and day 3 embryos.**
(TIF)

Figure S4 **Venn diagram representing the number of genes in each comparison and the overlaps between the three main comparison groups.** The day 3 embryo/MII oocyte/hESC signatures were defined as the intersection of the day 3 embryo signature (genes over-expressed in day3 embryos compared with TE samples; 1714 genes), the MII oocytes signature (genes over-expressed in MII oocytes compared with TE cells; 4444 genes ) and the hESC signature (genes up-regulated in hESC compared to TE samples, 5502 genes). The TE/MII oocyte/hESC signature were defined as the intersection of the TE signature (genes over-expressed in TE compared with day 3 embryos; 2196 genes), the MII oocyte signature (genes over-expressed in MII oocytes compared with day 3 embryos; 3198 genes ) and the hESC signature (genes over-expressed in hESCs compared with day 3 embryos; 8584 genes). The comparison between categories were generated by using the SAM software with a fold change ≥2 and FDR <1%.
(TIF)

Figure S5 **Expression of selected genes, which were up-regulated either in TE cells (** ***HSD3B1*** **,** ***HSD17B1*** **,** ***FDX1*** **,** ***PTGS*** **and** ***DNMT3L*** **) or day 3 embryos (** ***NANOG*** **,** ***MBD3L2*** **,** ***ZSCAN4*** **,** ***RFPL2*** **and** ***DPPA2*** **), and of beta-Actin in the panel of samples that includes MII oocytes and hESCs using the Amazonia! gene atlas explorer (** http://www.amazonia.transcriptome.eu **).** Abbreviations: hESC, human embryonic stem cell; hiPS, human induced pluripotent stem cells; TE, Trophectoderm; hFF, human foreskin fibroblasts; CNS, central nervous system; DT, digestive tract; H & L, Heart and muscle; HEMATO, various hematopoietic tissues; End, Endometrium; PL, placenta.
(TIF)

Table S1 List of the 1,714 transcripts specific to the day 3 embryo molecular signature.
(XLS)

Table S2 List of the 2,196 transcripts specific to the TE molecular signature.
(XLS)

Table S3 Primer pairs used for validating the array data by qRT-PCR.
(DOC)

Table S4 List of the transcripts included in the signatures analyzed in this manuscript. (a) Day 3 embryo/MII oocyte signature, (b) Day 3 embryo/hESC signature, (c) TE/MII oocyte signature, (d) TE/hESC signature, (e) specific day 3 embryo signature and (f) specific TE signature.
(XLS)

Table S5 List of the 104 genes that were up-regulated both in TE cells obtained by hESCs differentiation in the presence of BMP4 for 10 days (transcriptome analysis by Marchand et al, [29]) and in TE cells isolated from day 5 embryos (this study).
(XLS)
