# Supplementary figures and images for: Transcriptome Analysis during Human Trophectoderm Specification Suggests New Roles of Metabolic and Epigenetic Genes
Source: PLoS One. 2012 Jun 22;7(6):e39306. doi: 10.1371/journal.pone.0039306 (PMC3382239; doi:10.1371/journal.pone.0039306)

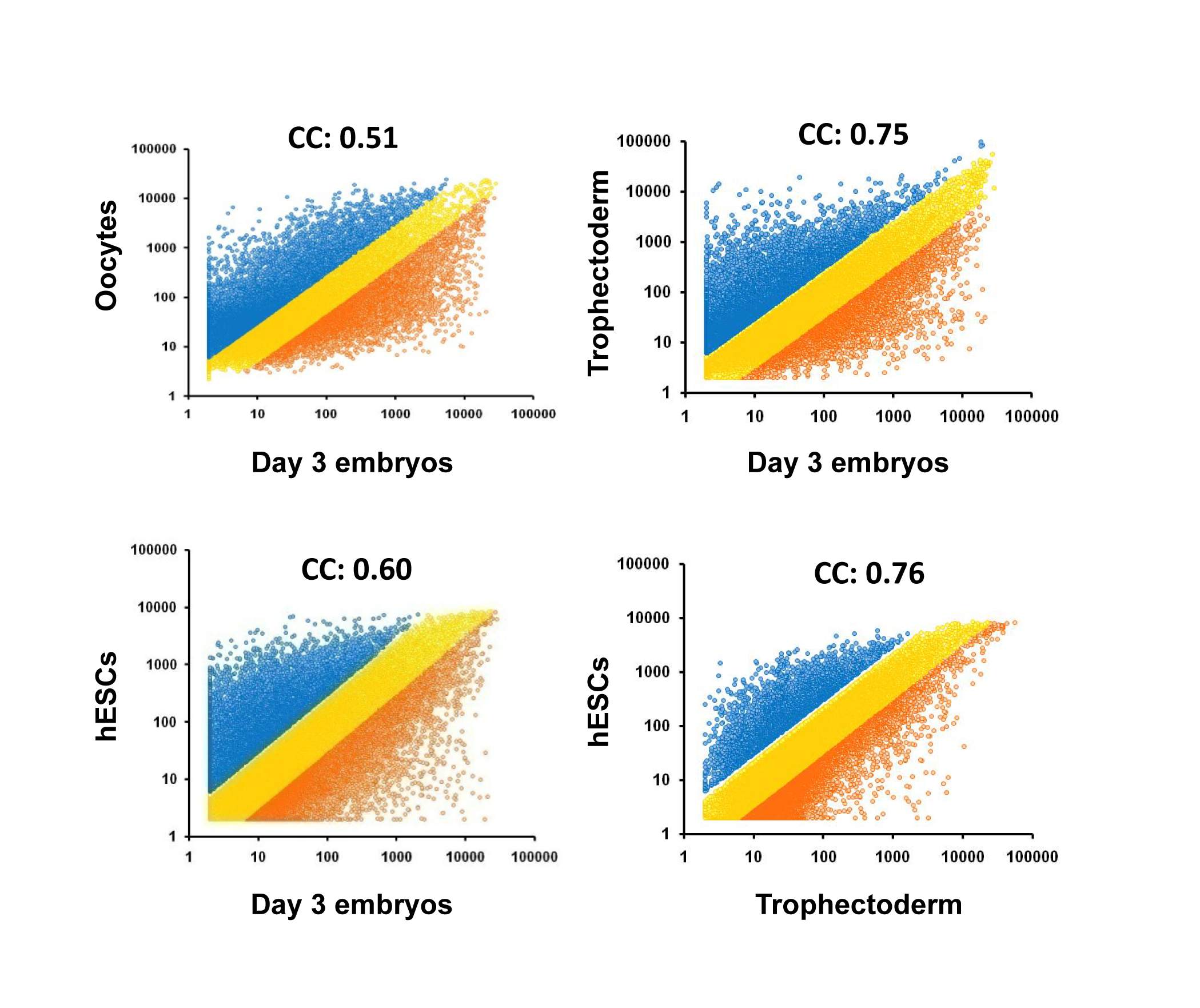

Supplement: Figure S1 — Scatter plots showing the comparative distribution of transcripts in mature MII oocytes, day 3 embryos, TE and hESC samples. Each sample was plotted against all the other samples to visualize expression variations. The blue areas highlight a greater than two-fold gene expression difference (up-regulated) between the X-axis and Y-axis samples. The orange areas indicate a greater than two-fold gene expression difference (down-regulated) between the X-axis and Y-axis samples. The yellow areas highlight a 0.5- to 2-fold gene expression difference between the X-axis and Y-axis samples. For each couple of samples, the Pearson’s correlation coefficient was computed (r). (TIF) [file pone.0039306.s001.tif]

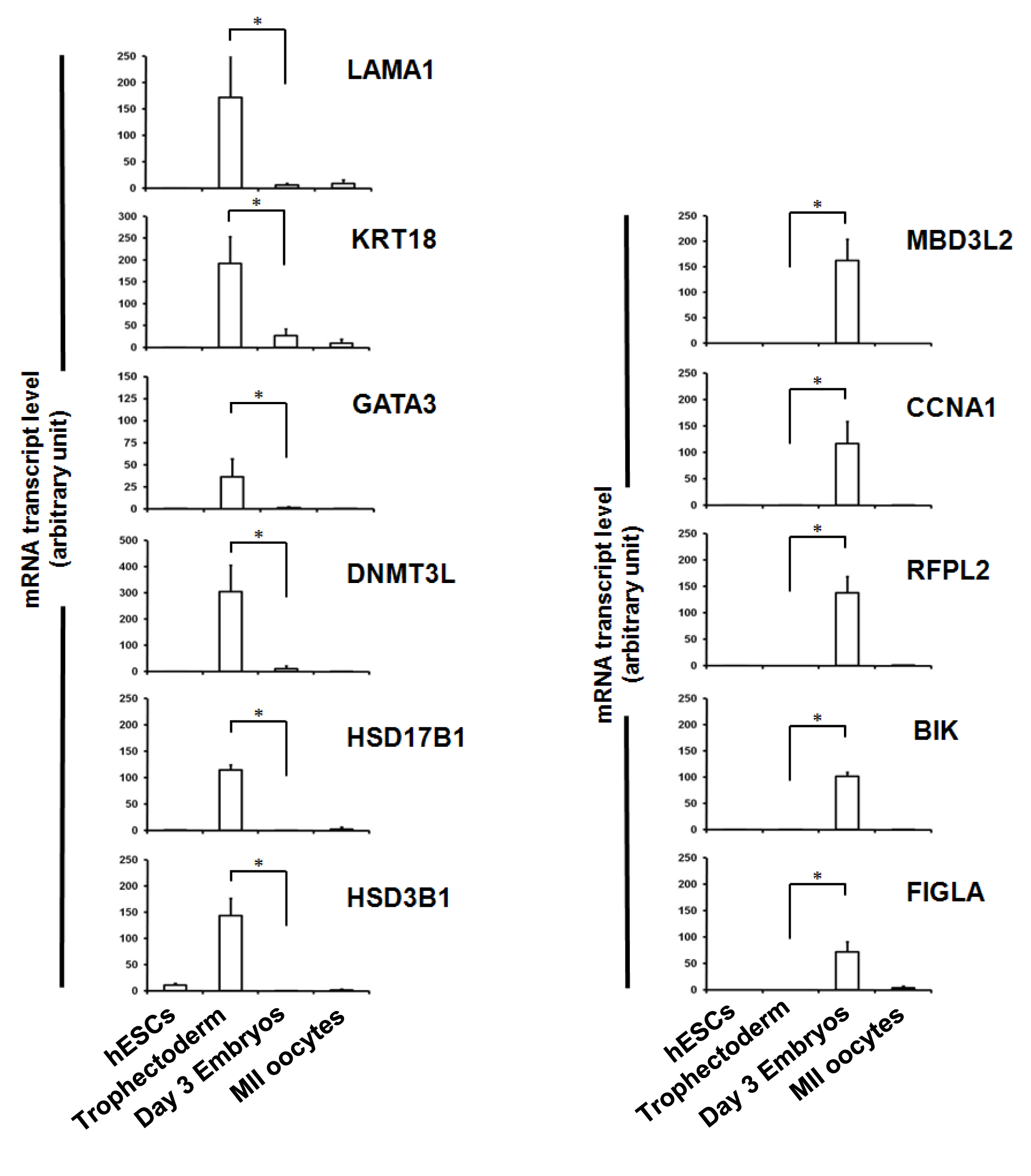

Supplement: Figure S2 — Quantitative RT–PCR validation of the microarray results: All qRT–PCR results were normalized to the expression of GAPDH in each sample and are the mean ± SEM of individual day 3 embryos (n = 3), TE (n = 3), pooled MII oocyte (n = 3) and hESC (n = 3) samples analyzed in duplicate. *P<0.05 was considered significant (TIF) [file pone.0039306.s002.tif]

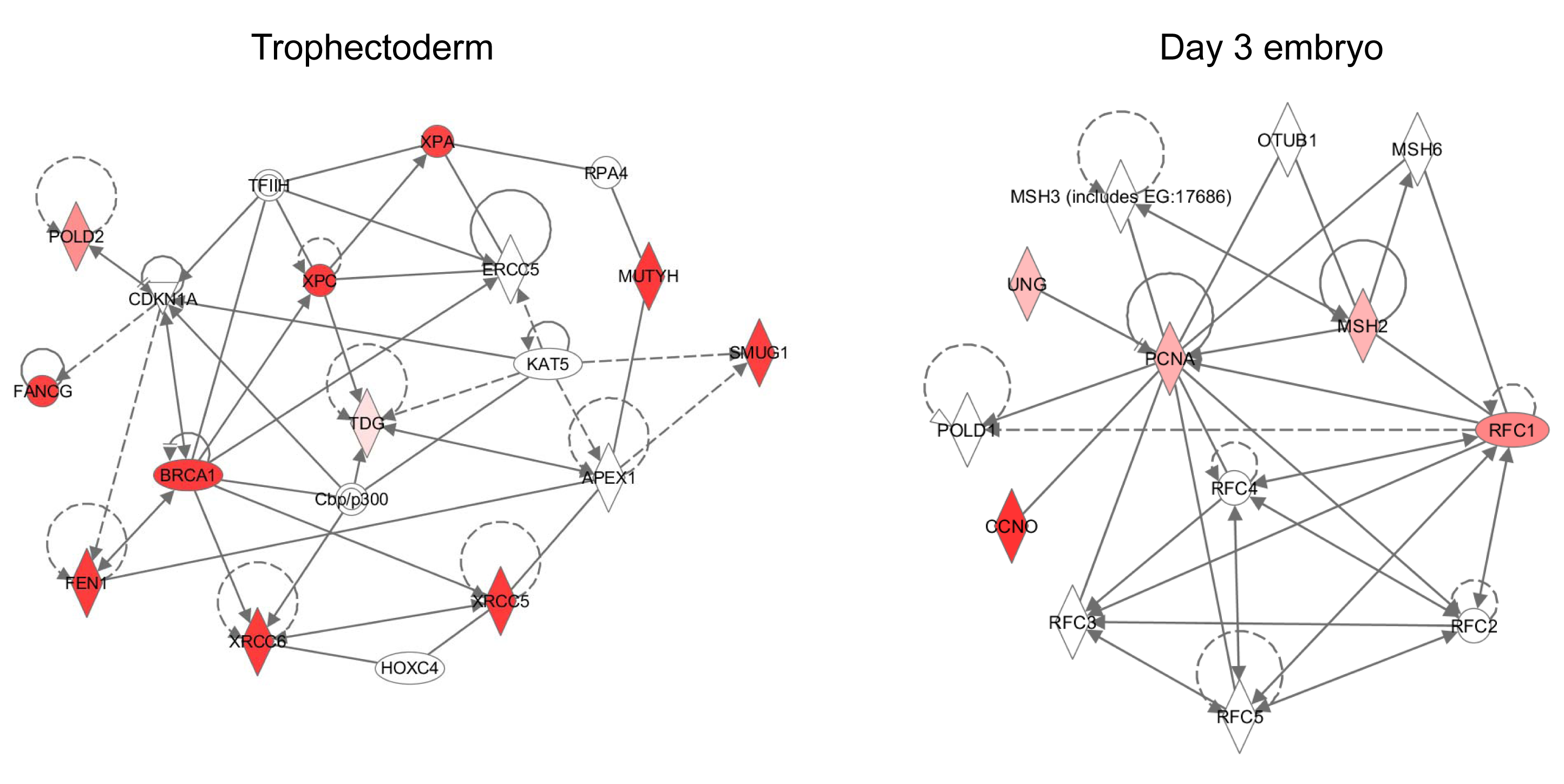

Supplement: Figure S3 — IPA results showing the network of DNA repair genes that are up-regulated in TE samples from day 5 human blastocysts and day 3 embryos. (TIF) [file pone.0039306.s003.tif]

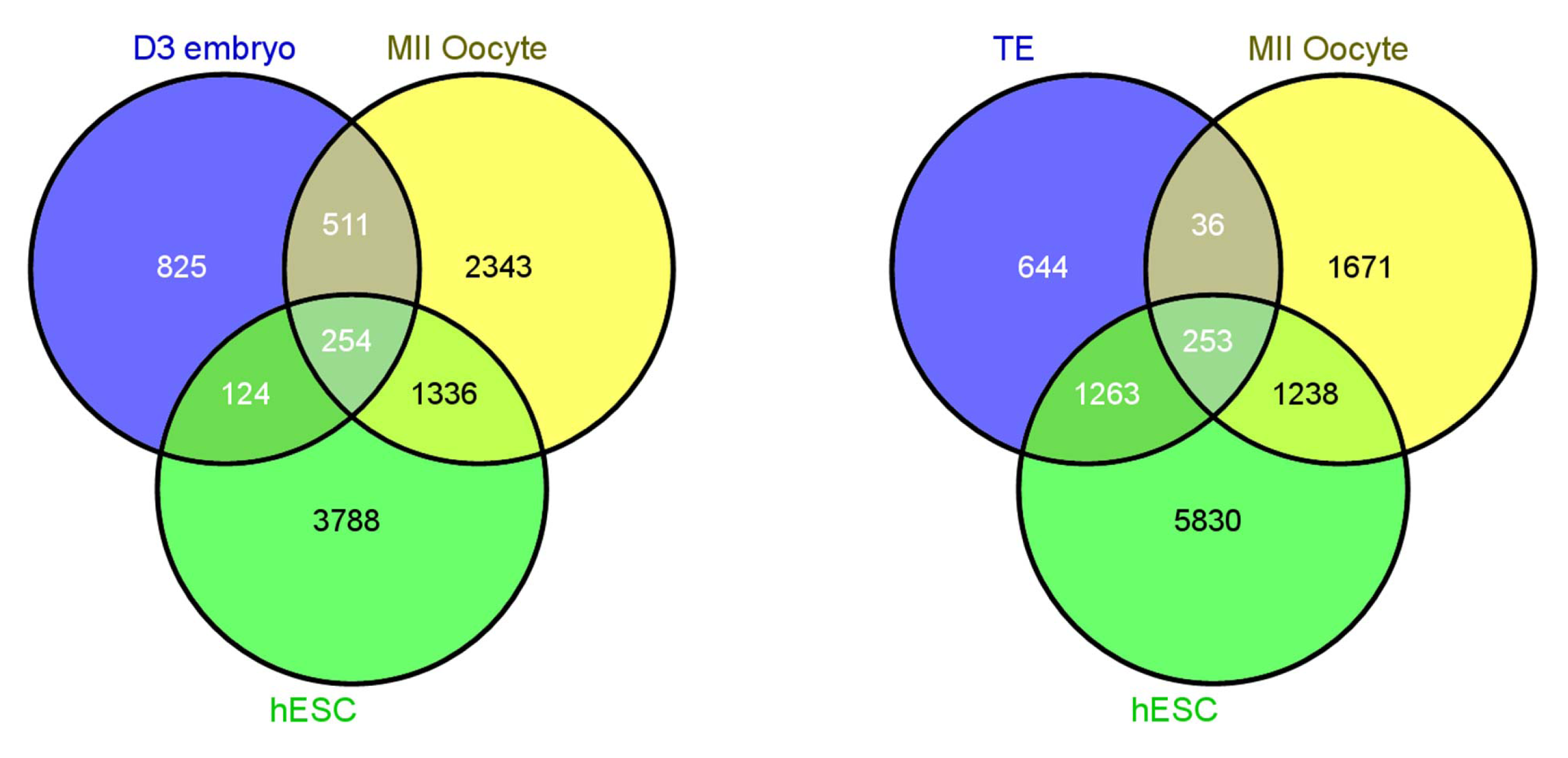

Supplement: Figure S4 — Venn diagram representing the number of genes in each comparison and the overlaps between the three main comparison groups. The day 3 embryo/MII oocyte/hESC signatures were defined as the intersection of the day 3 embryo signature (genes over-expressed in day3 embryos compared with TE samples; 1714 genes), the MII oocytes signature (genes over-expressed in MII oocytes compared with TE cells; 4444 genes ) and the hESC signature (genes up-regulated in hESC compared to TE samples, 5502 genes). The TE/MII oocyte/hESC signature were defined as the intersection of the TE signature (genes over-expressed in TE compared with day 3 embryos; 2196 genes), the MII oocyte signature (genes over-expressed in MII oocytes compared with day 3 embryos; 3198 genes ) and the hESC signature (genes over-expressed in hESCs compared with day 3 embryos; 8584 genes). The comparison between categories were generated by using the SAM software with a fold change ≥2 and FDR <1%. (TIF) [file pone.0039306.s004.tif]

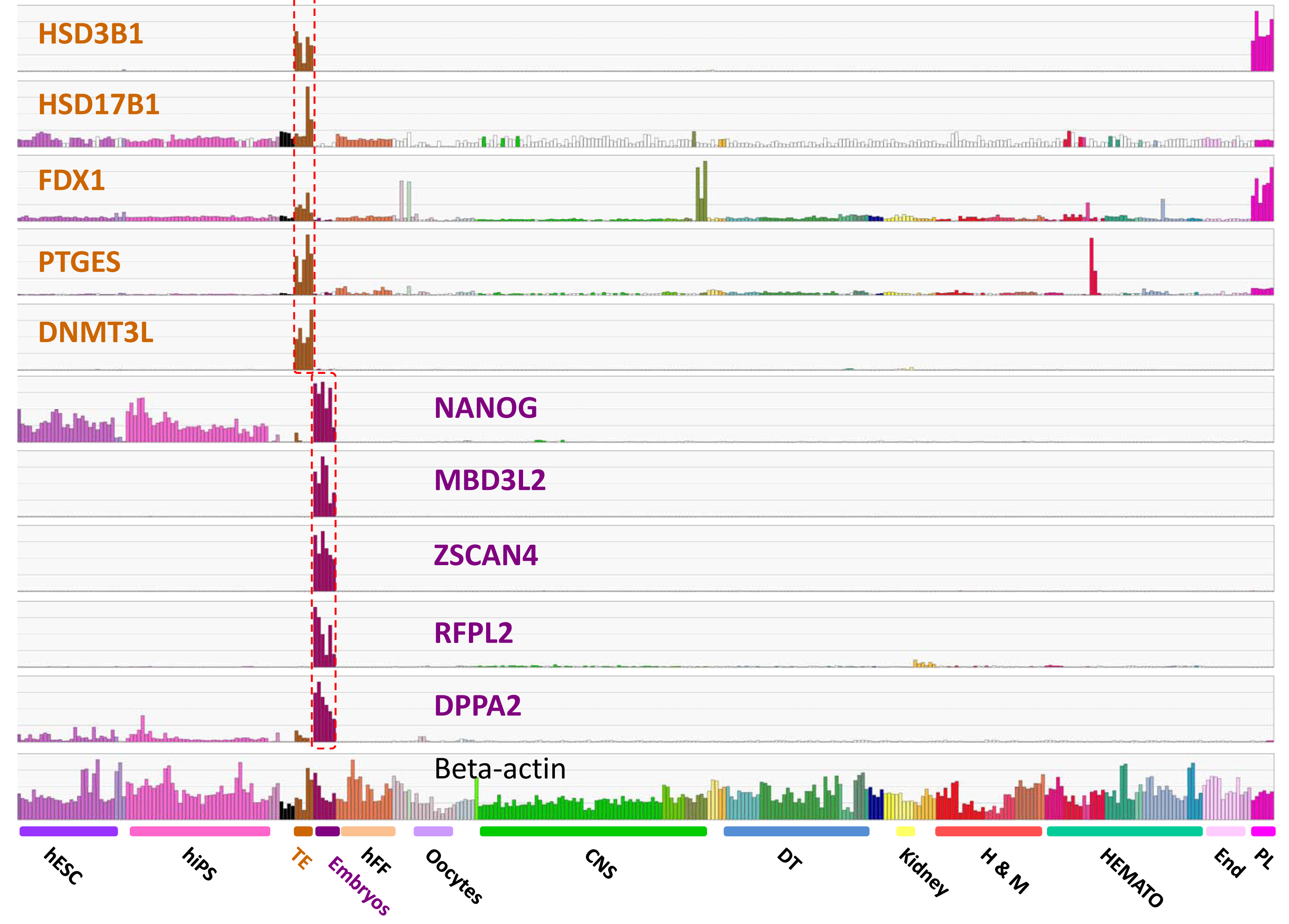

Supplement: Figure S5 — Expression of selected genes, which were up-regulated either in TE cells ( HSD3B1 , HSD17B1 , FDX1 , PTGS and DNMT3L ) or day 3 embryos ( NANOG , MBD3L2 , ZSCAN4 , RFPL2 and DPPA2 ), and of beta-Actin in the panel of samples that includes MII oocytes and hESCs using the Amazonia! gene atlas explorer ( http://www.amazonia.transcriptome.eu ). Abbreviations: hESC, human embryonic stem cell; hiPS, human induced pluripotent stem cells; TE, Trophectoderm; hFF, human foreskin fibroblasts; CNS, central nervous system; DT, digestive tract; H & L, Heart and muscle; HEMATO, various hematopoietic tissues; End, Endometrium; PL, placenta. (TIF) [file pone.0039306.s005.tif]
