# Supplementary material for: Transcriptome Analysis during Human Trophectoderm Specification Suggests New Roles of Metabolic and Epigenetic Genes
Source: PLoS One. 2012 Jun 22;7(6):e39306. doi: 10.1371/journal.pone.0039306 (PMC3382239; doi:10.1371/journal.pone.0039306)
Supplement: Table S3 — Primer pairs used for validating the array data by qRT-PCR. (DOC) [file pone.0039306.s008.doc]

**Table S4 : Primer pairs used for validating the array data by qRT-PCR**

| **Gene** | **Primer sequences (Forward, Reverse)** |
| --- | --- |
| **MBD3L2** | Forward: CGTTCACCTCTTTTCCAAGC |
|  | Reverse: TGACCTGGTTGTCAGGATGA |
|  |  |
| **CCNA1** | Forward: GCACCCTGCTCGTCACTTG |
|  | Reverse:CAGCCCCCAATAAAAGATCCA |
|  |  |
| **RFPL2** | Forward: ACCTGGGAGTCTGCAGAGAA |
|  | Reverse:ACATGGGAACCACTTTCAGC |
|  |  |
| **FIGLA** | Forward: AATCTCAACCGTGGTTTTGC |
|  | Reverse:CTTGCCGAGGATGTATGTGA |
|  |  |
| **LAMA1** | Forward: AGCGGATATGCAGCTCTTGT |
|  | Reverse:GCCGTCCACAAGCTCTAGTC |
|  |  |
| **BIK** | Forward: TCTGCAATTGTCACCGGTTA |
|  | Reverse: TTGAGCACTCCTGCTCCTC |
|  |  |
| **KRT18** | Forward: GAGTATGAGGCCCTGCTGAACATCA |
|  | Reverse: GCGGGTGGTGGTCTTTTGGAT |
|  |  |
| **GATA3** | Forward: TGCAGGAGCAGTATCATGAAGCCT |
|  | Reverse: GCATCAAACAACTGTGGCCAGTGA |
|  |  |
| **DNMT3L** | Forward: GTGGTTGATGTCACAGACAC |
|  | Reverse: AACATCCAGAAGAAGGGCCT |
|  |  |
| **HSD3B1** | Forward: TCCATACCCACACAGCAAAA |
|  | Reverse: GTTGTTCAGGGCCTCGTTTA |
|  |  |
| **HSD17B1** | Forward: TTCCTGCCAGACATGAAGAGGC |
|  | Reverse: AGAACCGCCAGACTCTCGCATA |
